# Die „Witzelsucht“. Über die Einführung und die Bedeutung des Begriffs von Hermann Oppenheim im Jahr 1890 und seine Abgrenzung zur „Moria“

**DOI:** 10.1007/s00115-024-01668-8

**Published:** 2024-05-14

**Authors:** Nina C. Moenikes, Maria Strauß, Holger Steinberg

**Affiliations:** 1 Forschungsstelle für die Geschichte der Psychiatrie, Klinik und Poliklinik für Psychiatrie und Psychotherapie, Medizinische Fakultät, Universität Leipzig, Leipzig, Deutschland; 2 Klinik und Poliklinik für Psychiatrie und Psychotherapie, Universitätsklinikum Leipzig AöR, Leipzig, Deutschland

**Keywords:** Pathologischer Humor, Hemisphärendominanz, Moritz Jastrowitz, Frontalhirn, Hirntumor, Brain tumor, Cerebrale Dominanz, Moritz Jastrowitz, Frontal lobe, Pathological humor

## Abstract

Hermann Oppenheim (1858–1919), zu seiner Schaffenszeit um 1900 ohne akademische Karriere gebliebener deutscher Nervenarzt, hat sich durch seine Beiträge zur multiplen Sklerose, Syphilis und die allerdings kontrovers diskutierte Studie über die traumatische Neurose schon zu Lebzeiten einen Namen gemacht. Nahezu unbekannt ist hingegen, dass der heute noch international verwendete Begriff „Witzelsucht“ 1890 von ihm eingeführt wurde. Moritz Jastrowitz beschäftigte sich praktisch zeitgleich mit Verhaltensauffälligkeiten aufgrund von Stirnhirnverletzungen und benutzte den Begriff „Moria“ für eine Form von Geistesstörung, die mit einer Art kindischen Benehmens und unangemessener Heiterkeit einhergeht. Oppenheim sah das kritisch, grenzte seine „Witzelsucht“ davon ab. Er wollte mit diesem Terminus in einem viel engeren Sinne humoristische Schwachsinnigkeit bezeichnen, die frappant konträr zur übrigen Leidenssymptomatik bei Großhirngeschwülsten stehe. Oppenheim erkannte vor allem das rechte Frontalhirn als wichtige funktionelle Einheit für humoristische Verhaltensweisen. Die moderne Forschung bestätigt, dass die Verarbeitung von Humor eine komplexe Interaktion mehrerer Hirnregionen erfordert und dass insbesondere die Schädigung des rechten Frontallappens zur Störung „Witzelsucht“ führen kann. Ob eine gleichzeitige Schädigung der linken Hemisphäre vorliegen muss oder dies in Abhängigkeit zur individuellen Hemisphärendominanz zu setzen ist, bedarf weitergehender Forschung.

## Abstract (en)

Hermann Oppenheim (1858–1919) was a German neurologist without an academic career, who in his productive period around 1900 made a name for himself during his lifetime as a major player in the history of German neurology with his many contributions to multiple sclerosis, syphilis and the controversial study of traumatic neurosis; however, it is almost unknown that in 1890 he introduced the term “witzelsucht”, which is still used internationally today. Moritz Jastrowitz dealt with behavioral abnormalities due to frontal brain injuries 1 year earlier and used the term “moria” for a form of mental disorder associated with a kind of childish behavior and inappropriate jocularity. Oppenheim was critical of this and differentiated his “witzelsucht” from this. With this term he wanted to describe humoristic feeble-mindedness in a much narrower sense, which stands in striking contrast to the usual symptoms in cases of cerebral tumors. Oppenheim recognized the frontal brain, particularly the right brain, to be an important functional unit for humorous behavior. Modern research has confirmed that the processing of humor requires a complex interaction of multiple brain regions. Damage to the right frontal lobe or to connecting structures can lead to the disorder “witzelsucht”. Whether a simultaneous damage to the left hemisphere must be present or if this is dependent on the individual dominant hemisphere, needs further research.

## Witzelsucht

Die „Witzelsucht“ ist eine heute noch verwendete Wortneuschöpfung des Nervenarztes Hermann Oppenheim aus dem Jahre 1890 [18, 22]. Der Begriff scheint seine Prägnanz eigentlich im Namen zu tragen und doch kommt er in der heutigen Literatur nicht einheitlich definiert zur Anwendung.

Dieser Artikel setzt sich mit Oppenheims Ausführungen über die „Witzelsucht“ auseinander, vergleicht diese mit dem oft synonym verwendeten Terminus „Moria“ und thematisiert die heutige Relevanz beider Konzepte. Zudem werden die neurowissenschaftlichen Ursachentheorien des Symptoms der „Witzelsucht“ betrachtet.

## Oppenheim: vom Musterschüler aus Warburg zum bekannten, aber umstrittenen Nervenarzt

Hermann Oppenheim, 1857 in der westfälischen Kleinstadt Warburg als Sohn jüdischer Eltern geboren, absolviert mit herausragenden Leistungen seine Schulausbildung und entscheidet sich nach dem Abitur für ein Studium der Medizin in Göttingen und Bonn. Nach kurzzeitiger Tätigkeit am Maison de Santé in Berlin-Schöneberg folgt er 1883 dem Ruf seines späteren Mentors Carl Westphal (1824–1900) an die Charité und entdeckt im Zuge dessen seine Passion für die Neurologie [21]. Trotz Oppenheims Engagement in Forschung und Lehre wird sein Antrag auf ein Extraordinariat in den Folgejahren abgelehnt. Hierbei ist anzunehmen, dass antisemitische Vorbehalte bei dieser Entscheidung eine Rolle spielten [8]. Nach Westphals Tod zieht er sich resigniert in seine Privatklinik in Berlin am Schiffbauerdamm und in seine privaten Forschungen zurück und widmet sich seinen zahlreichen Studien, welche bis heute noch Anerkennung finden [8]. Zu diesen gehört auch sein 1894 veröffentlichtes *Lehrbuch der Nervenkrankheiten* [20], das sich bis zur 7. Auflage 1923 weltweit als ein Standardwerk in der Neurologie etabliert [2]. Die Forschung zur „traumatischen Neurose“ [19] ist bis heute untrennbar mit dem Namen Oppenheim verknüpft, doch war es gerade diese, die ihm am Ende seines Wirkens viel Kritik entgegenbrachte. Oppenheim war überzeugt, dass neurotische Symptome durch Mikroverletzungen im Gehirn und nicht wie allgemein angenommen durch Willensschwäche verursacht würden. Diese These findet in der Fachwelt seiner Zeit wenig Zustimmung und wirft einen Schatten auf seine letzten Schaffensjahre [11].

Mit seinen über 300 Schriften zu unterschiedlichen Themen aus Medizin und Gesellschaft ist Hermann Oppenheim einer der großen Charaktere in der Geschichte der deutschen Nervenheilkunde [8]. Zu diesen gehört auch seine Arbeit „Zur Pathologie der Großhirngeschwülste“ [18], in der er den Neologismus „Witzelsucht“ 1890 erstmalig verwendet und beschreibt.

## Das unerforschte Frontalhirn

Der Fall von Phineas Gage, bekannt als „Crowbar Case“, prägt die Geschichte der Neurowissenschaften und betont erstmals das Frontalhirn als funktionell bedeutendes Areal in der öffentlichen Wahrnehmung [15]. Gage erlitt 1848 bei einem Arbeitsunfall eine penetrierende Verletzung des linken Frontallappens durch eine Eisenstange. Er erholte sich erstaunlich schnell und zeigte keine langfristigen motorischen oder kognitiven Beeinträchtigungen. Auffällig war jedoch eine Veränderung seiner Persönlichkeit [15, 24]. Gerade auch dieser Fall offenbart, dass die Forschungserkenntnisse zu diesem Teil des Gehirns selbst in der Diskussion am Ende des 19. Jahrhunderts noch sehr begrenzt waren [26]. Carl Wernicke (1848–1905) schreibt 1881, dass Schädigungen im Bereich des Stirnlappens meist symptomlos blieben und Charakterveränderungen keiner speziellen Lokalisation zugeordnet werden könnten [27].

Martin Bernhardt (1844–1915) postuliert, dass es nicht möglich sei, Formen von Geistesstörungen mit der Entwicklung von Tumoren in bestimmten Hirnregionen in Verbindung zu bringen. Er ergänzt: „nur das kann man vielleicht sagen, dass es scheint, als ob die Entwicklung von Tumoren in der vorderen Schädelgrube (…) symptomatisch in einer ganz besonderen Art kindischen Benehmens und Sprechens neben abnormaler Schlafsucht zum Ausdruck kommt“ [1, S. 11–12].

Kontrastierend dazu mutmaßt 1894 der italienische Neurologe Leonardo Bianchi (1848–1927), ob das Frontalhirn den Sitz der Intelligenz darstelle oder nur ein weiteres motorisches Zentrum sei [3]. 1888 verfasst Leonore Welt (1859–1944) einen Beitrag zur Rolle und Funktion der vorderen Hirnabschnitte. In ihrer beeindruckenden Zusammenstellung von Kasuistiken „Über Charakterveränderungen des Menschen infolge von Läsionen des Stirnhirns“ betont sie die Bedeutung des Frontalhirns für das Wesen und die Psyche des Menschen. Im Rahmen dessen macht sie die Beobachtung, dass einige Patienten mit Schädigung des rechten Stirnlappens einen Hang zur Äußerung schlechter Witze besäßen [26].

Moritz Jastrowitz (1839–1912), ein Berliner ideengeschichtlicher Wegbereiter Oppenheims, veröffentlicht im selben Jahr seine Abhandlung „Beiträge zur Localisation im Grosshirn und über deren praktische Verwerthung“ [12]. In dieser beschäftigt er sich mit derselben Thematik wie Welt, gelangt auch zu ähnlichen Schlüssen und verwendet den Begriff „Moria“, um eine Form der Geistesstörung zu beschreiben, die mit Blödsinn und eigentümlich heiterer Aufregung einhergehe und nur bei Tumoren oder anderen Schädigungen des Frontallappens auftrete. Dieses eigentümliche Verhalten sei pathognomonisch für die Lokalisation im Stirnhirn [12]. Jastrowitz schreibt über dieserart betroffene Patienten: „Vielleicht lag diese unfreiwillige Komik in dem Contrast zwischen ihrer sonstigen Apathie und Vergesslichkeit und plötzlichen Lichtblicken, in denen sie, wahrscheinlich ihrem ursprünglichen Naturell entsprechend, muntere und selbst sarkastische Äußerungen thaten“ [12, S. 34].

## Oppenheims Beobachtungen und sein Neologismus „Witzelsucht“

Oppenheim greift im Folgejahr die Beobachtungen seines Kollegen Jastrowitz auf und stellt in seiner dreiteiligen Abhandlung „Zur Pathologie der Großhirngeschwülste“ [18] 23 Kasuistiken von Patienten mit Hirntumoren und deren Symptomen zusammen. Als charakteristisch und mit „hohem diagnostischen Werth“ für Neubildungen im Gehirn beschreibt er bei seinen Patienten vorrangig die Schlafsucht und Benommenheit [18, S. 43]. Ergänzend dazu bemerkt er anfänglich häufig auftretende psychische Anomalien. Oppenheim stellt zur Diskussion, ob „Schwermuth“, „Todesfurcht“, „Reizbarkeit“ als Resultat der sich anbahnenden Aphasie zu sehen seien oder ein eigenständiges, durch den Tumor bedingtes Symptom darstellten [18, S. 35–36]. Hiervon grenzt er jene Patienten ab, die im Verlauf anstelle oder zusätzlich zu den beschriebenen Negativsymptomen auch eine Positivsymptomatik mit Euphorie und – wie er sie nun erstmals nennt – mit typischer „Witzelsucht“ [18, S. 36] zeigten.

Dabei weist Oppenheim auf die Krankengeschichte von vier Patienten hin: Diese präsentieren sich mit einem Hang zur Witzelei, der sich konträr zur Gesamtsituation darstellt. Er schildert hier beispielhaft den Kasus eines 80-jährigen Mannes, der zunächst mit „klassischen“ Symptomen wie Kopfschmerzen, Erbrechen, Visusminderung sowie zunehmender Somnolenz auffällt. Der Berliner Arzt schreibt: „Der Patient ist somnolent, antwortet träge und wie im Halbschlaf. Ausgesprochene psychische Anomalien fehlen, nur macht sich in den Aeusserungen des Patienten eine im Widerspruch zu dem schweren Leiden stehende Heiterkeit geltend. Beispiel: Nach seinem Befinden gefragt, äussert er: Na, Herr Doctor! Wie solls gehen? Der Kopf ist immer noch oben! Wie gehts Ihnen denn, Herr Doctor? Auch auf zwei Beinen, nicht wahr? etc. Diese Geschwätzigkeit ist umso auffallender bei einem fortdauernd im Halbschlaf liegendenden Individuum, das spontan kaum eine Aeusserung hören lässt und sobald sich der Arzt vom Krankenbett entfernt hat, wieder in seinen Schlummer verfällt.“ Post mortem habe sich ein Gliom im rechten Stirnlappen gezeigt [18, S. 706]. Drei weitere Patienten zeigen laut Oppenheim ebensolches Verhalten, das von humoristischer Schwachsinnigkeit zeuge und sich konträr zur übrigen Symptomatik respektive der Schwere ihres Leidens präsentiere. Der Humor sei unpassend und teils spottender Natur [18, S. 710]. In all diesen Fällen habe sich bei der Obduktion eine Raumforderung im rechten Frontallappen gezeigt. Oppenheim mutmaßt daher: „Suchen wir nach dem Sitz des Tumors in den Fällen, die durch diese Erscheinung ausgezeichnet sind, so springt allerdings die Thatsache in die Augen, dass es sich in den vier Fällen um Tumoren des rechten Stirnlappens handelt (…). Wenn wir sie bei den Kranken, die einen Tumor ausschließlich im linken Stirnlappen trugen, vermissen, so liegt der Grund zunächst darin, dass diese fast alle aphasisch waren.“ [18, S. 58]. Diese von ihm beobachtete Neigung zur Witzelei benennt er selbst „Witzelsucht“, auch um eine Abgrenzung zu der ein Jahr zuvor durch Jastrowitz verwendeten Bezeichnung „Moria“ zu schaffen. Oppenheim übt Kritik an der von Jastrowitz benutzten Terminologie und stellt deren Nutzen für die Hirnforschung infrage. Zwar sieht dieser die von Jastrowitz beobachteten Wesensveränderungen ebenfalls als relevant an, doch betrachtet Oppenheim den Begriff „Moria“ als zu weit gefasst und unklar definiert. Es sei ihm daraus nicht ersichtlich, welche Bedeutung sein Kollege dieser Erscheinung beimesse, da dieser sowohl die „läppische Art“ [18, S. 56] als auch die Negativstimmung seiner Patienten unter „Moria“ subsumiere. Das humoristische Verhalten werde zwar als pathognomonisch gewertet, allerdings im Verlauf durch Jastrowitz wieder relativiert, indem er dies als kurz durchblickenden natürlichen Charakter der Patienten zu erklären versuche. Aufgrund dieser uneindeutigen Wertung sieht Oppenheim den Terminus „Moria“ als nicht verwertbar für weiterführende Forschungen an [18, S. 56].

## „Witzelsucht“ vs. „Moria“: ein Vergleich

Oppenheim und Jastrowitz verliehen in historischer Rückschau jeweils den Begriffen „Witzelsucht“ und „Moria“ Bedeutung und Kontext. Oppenheim legte den Fokus auf das spezifische Symptom der ungehemmten und unangemessenen Witzelei. Er betrachtete es als ein Zeichen für eine mögliche Schädigung des Cerebrums, insbesondere des rechten Stirnhirnlappens. Jastrowitz hingegen verwendete den aus dem Altgriechischen stammenden Begriff „Moria“ für einen Symptomenkomplex, der durch eine Vielzahl von Verhaltensänderungen gekennzeichnet ist und über den rein humoristischen Aspekt hinausgeht. Die durch ihn beschriebene Symptomatik stellt sich noch vielfältiger als die von Oppenheim aufgezeigte dar. So wies Jastrowitz darauf hin, dass seine Patienten ebenfalls „zu groben Späßen und Neckereien aufgelegt“ [12, S. 34] seien, dies paare sich allerdings mit Böswilligkeit und geistiger Schwäche. Auch könnten erotische Züge sowie Verwirrung beobachtet werden. Während Jastrowitz „Moria“ der Schädigung des Frontallappens zuschreibt, geht Oppenheim einen Schritt weiter und betont, dass er auffälligerweise die „Witzelsucht“ seiner Patienten nur bei primärer Beteiligung des rechten Frontallappens beobachte [18].

Das Wort „Moria“ bedeutet übersetzt etwa „Dummheit“ oder „Torheit“ [10]. Entgegen der häufig zu lesenden Behauptung in heutigen Publikationen [7, 14, S. 16] wurde der Begriff „Moria“ nicht erstmals durch Jastrowitz im Jahre 1888 in dieser Bedeutung in die Medizin eingeführt. Bereits 1844 ist der Terminus „Moria“ im Kritisch-etymologisch-medicinischen Lexikon von Krause aufgeführt. Dort wird dieser mit „Einfalt“, „Stumpfsinn“ und „Blödsinn“ übersetzt [13, S. 634]. Auch im englischen Sprachraum ist „Moria“ in der Medizin schon 40 Jahre vor Jastrowitz’ Arbeit bekannt als Beschreibung für „Idiotism“, „Demens“, „Amnesia“ [6, S. 557]. Diese Begriffe und insbesondere die mit ihnen verbundenen negativen Konnotationen spiegeln sich in Jastrowitz’ Verwendung im Hinblick auf seine Patienten wider. „Witzelsucht“ im Kontrast dazu fokussiert den humoristischen Aspekt der Verhaltensauffälligkeit. Oppenheim stellt durch seine „Witzelsucht“ keine „Blödsinnigkeit“ heraus, sondern den reinen Hang zur Witzelei, die in gegebenen Situationen als unpassend oder unangemessen anmuten kann, doch nicht durch die allgemeine Verwirrung der Patienten erklärbar ist. Vorrangig ist es der Kontrast zwischen dem Positivsymptom des hervorstechenden Humors und der zugrunde liegenden schweren Pathologie, der von Oppenheim in den Fokus gerückt wird. „Moria“ hingegen beschreibt eine allgemeine Geistesstörung, die mit vielseitiger Symptomatik einhergeht.

## Die Bedeutung des rechten Stirnlappens

Oppenheim mutmaßt, dass prädominant eine Schädigung des rechten Stirnlappens zur „Witzelsucht“ führt [18]. Mit dieser Mutmaßung kongruent stellt sich die aktuelle Forschungshypothese zu diesem Thema dar. Um zu evaluieren, ob das Auftreten von „Witzelsucht“ einer bestimmten Hirnlokalisation zuzuordnen ist, ist es von Relevanz, zunächst den Humor als ein komplexes kognitives Konstrukt zu definieren. Er ist eines der wichtigsten sozialen Phänomene der menschlichen Interaktion und ein entscheidender Bestandteil in der Bildung interpersoneller Beziehungen [5, 23]. Die Forschung hat gezeigt, dass Humor eines vielschichtigen Zusammenspiels multipler kognitiver Zentren und Verknüpfungen bedarf [23]. Dahingehend sind mehrere Aspekte zu betrachten: Der erste Aspekt umfasst die kognitive Kompetenz, in der Lage zu sein, einen Witz inhaltlich zu verstehen. Dies erfolgt durch die Wahrnehmung von Inkongruenz beziehungsweise Unvereinbarkeit zwischen einerseits einer erwarteten Perspektive bzw. Vorahnung und andererseits der Pointe. Die Überraschung löst dann eine Suche nach alternativen Erklärungen und die Auflösung der Inkongruenz aus. Zweitens erfolgt nach dem Empfinden des Humors dessen eigentliche Verarbeitung. Dieser Prozess involviert die Aktivität dopaminerger Lust- und Belohnungszentren, speziell des ventralen Striatums und des Nucleus accumbens. Die frontalen Hirnregionen sind entscheidend an der Erkennung und Auflösung von Inkongruenzen beteiligt, wobei der linke Frontallappen tendenziell auf einfache humorvolle Reize reagiert, während der rechte Frontallappen eher bei der Verarbeitung komplexeren Humors aktiv ist. Des Weiteren können auch andere Hirnregionen zur Wahrnehmung und Auflösung von Inkongruenzen beitragen, darunter der temporoparietale Übergang, der Precuneus, der posteriore zinguläre Kortex und der Gyrus parahippocampalis [7, 9]. Sobald die Inkongruenzen erfolgreich aufgelöst sind, werden emotionale Reaktionen ausgelöst, die als Heiterkeit empfunden werden und mit angenehmen emotionalen Erfahrungen einhergehen [17]. Das Frontalhirn stellt hier das integrative Zentrum durch Verbindung zu den benannten Hirnregionen dar und ist daher maßgebend am Verständnis, der Beurteilung und Produktion von Humor beteiligt [5, 7, 16]. Insbesondere der rechte Frontallappen spielt eine entscheide Rolle in der Entstehung von Humor [17, 23, 24]. Shammi und Stuss vergleichen in ihrer Forschung Patienten mit rechts- und linksseitiger Frontalhirnschädigung und stellen fest: „The ability of the right frontal lobe may be unique in integrating cognitive and affective information, an integration relevant for other complex human abilities, such as episodic memory and selfawareness“ [23]. Patienten mit einer Schädigung der rechten Seite zeigen ein erhaltenes Verständnis für den Überraschungsmoment, sind aber defizitär in Bezug auf das inhaltliche Verständnis, die Kohärenz des Witzes [23]. Es steht zur Diskussion, ob neben der rechtsseitigen Schädigung auch eine zusätzliche Dysfunktion der linken Seite nötig ist, um „Witzelsucht“ zu erzeugen [17]. Vardi und Kollegen implizieren ebenfalls die Bedeutung der rechten Hemisphäre für die Entstehung von „Witzelsucht“, beobachten aber, dass eine Schädigung in der subdominanten Hemisphäre zu Verhaltensmustern führt, die Einfluss auf die Verarbeitung des Humors haben [25]. Ob und inwieweit die Entstehung von Humor abhängig von der Hemisphärendominanz ist, bedarf weiterer Forschung. Der Frage, was nun aber zur pathologischen Witzelei führt, widmen sich Granadillo und Mendez. Sie sehen als Ursache ein Disinhibitionssyndrom, resultierend aus der Disruption von Faserverbindungen vom orbitofrontalen Kortex (OFC) zum anterioren zingulären Kortex (ACC). Die Schädigung des rechten Frontallappens führt zu reduzierter Sensibilität für extern generierten Humor, und die Enthemmung in Bezug auf den selbst generierten Humor ist Folge der fehlenden Inhibition im OFC-ACC-Kreislauf [7, 9].

## „Witzelsucht“ und „Moria“ in der heutigen Literatur

Heutzutage werden beide Begriffe, „Witzelsucht“ und „Moria“, international zur Bezeichnung der beschriebenen spezifischen Charakterveränderungen und damit assoziierten Hirnschädigungen verwendet. Meistens erfolgt die Verwendung synonym. Dies ist als problematisch zu erachten, da sie schon von Anfang an von Jastrowitz und Oppenheim in ihrer genauen Definition v. a. hinsichtlich der Ausgestaltung ihres Umfangs unterschiedlich angelegt waren. Während „Moria“ die „Witzelsucht“ hyperonymisch mit einschließt, bezieht sich Oppenheims Neologismus ausschließlich auf die humoristische Verhaltensauffälligkeit. Im Rahmen dieser falsch verstandenen Synonymität wurden „Moria“ respektive „Witzelsucht“ indessen längst nicht nur bei Patienten mit tumorösen Erkrankungen beobachtet, wie eingangs von Jastrowitz und Oppenheim. Wenige Beispiele mögen genügen: Mendez beschrieb 2005 einen Fall von „Witzelsucht“ und „Moria“ bei einer an frontotemporaler Demenz (FTD) erkrankten Patientin. Er postulierte, dass diese Symptomatik sich sogar als eine der prägnantesten und prominentesten bei der FTD darstellen könne. Darüber hinaus stellte er fest, dass häufig eine Schädigung der rechten anterioren orbitofrontalen Region ursächlich für diese sei [16]. Auch Patienten mit Neurosyphilis und Hirnblutungen können „Witzelsucht“ oder „Moria“ zeigen [16]. In dem von Chen beschriebenen Fall wurde ein 56-jähriger Mann als Folge einer Hirnblutung im rechten Putamen durch Hypersexualität und „Witzelsucht“ auffällig. Chen mutmaßte hinsichtlich der konkreten Ursache, dass durch die Blutung Verbindungsfasern unter anderem zum orbitofrontalen Kortex beschädigt seien [4].

## Fazit für die Praxis

Der deutsche Nervenarzt Hermann Oppenheim prägte 1890 den Terminus „Witzelsucht“. Er wollte damit die humoristische Schwachsinnigkeit bezeichnen, die frappant konträr zur übrigen Leidenssymptomatik bei Großhirngeschwülsten stehe und die er richtigerweise als die Folge einer Schädigung des rechten Frontallappens ansah. Moritz Jastrowitz beschäftigte sich fast zeitgleich mit derartigen Phänomenen und prägte den Begriff „Moria“ für eine Form von Geistesstörung, die mit „kindischem“ Benehmen und unangemessener Heiterkeit einhergeht. Eine Differenzierung der Begriffe scheint für die heutige Diagnose und Beschreibung sinnvoll, wird aber kaum beachtet. Oppenheims Arbeit weist weiter auf die mögliche Bedeutung des Humors bei pathologischen Verhaltensweisen hin, die im Rahmen verschiedenster Syndrome auftreten können. Dessen genaue Evaluation kann zu einer frühzeitigeren und genaueren Diagnose beitragen.
